# Supplementary material for: MHC1-TIP enables single-tube multimodal immunopeptidome profiling and uncovers intratumoral heterogeneity in antigen presentation
Source: bioRxiv. 2025 Jul 21:2025.07.17.664894. Preprint. [Version 1] doi: 10.1101/2025.07.17.664894 (PMC12330465; doi:10.1101/2025.07.17.664894)
Supplement: Supplement 1 [file media-1.pdf]

Supplementary figures

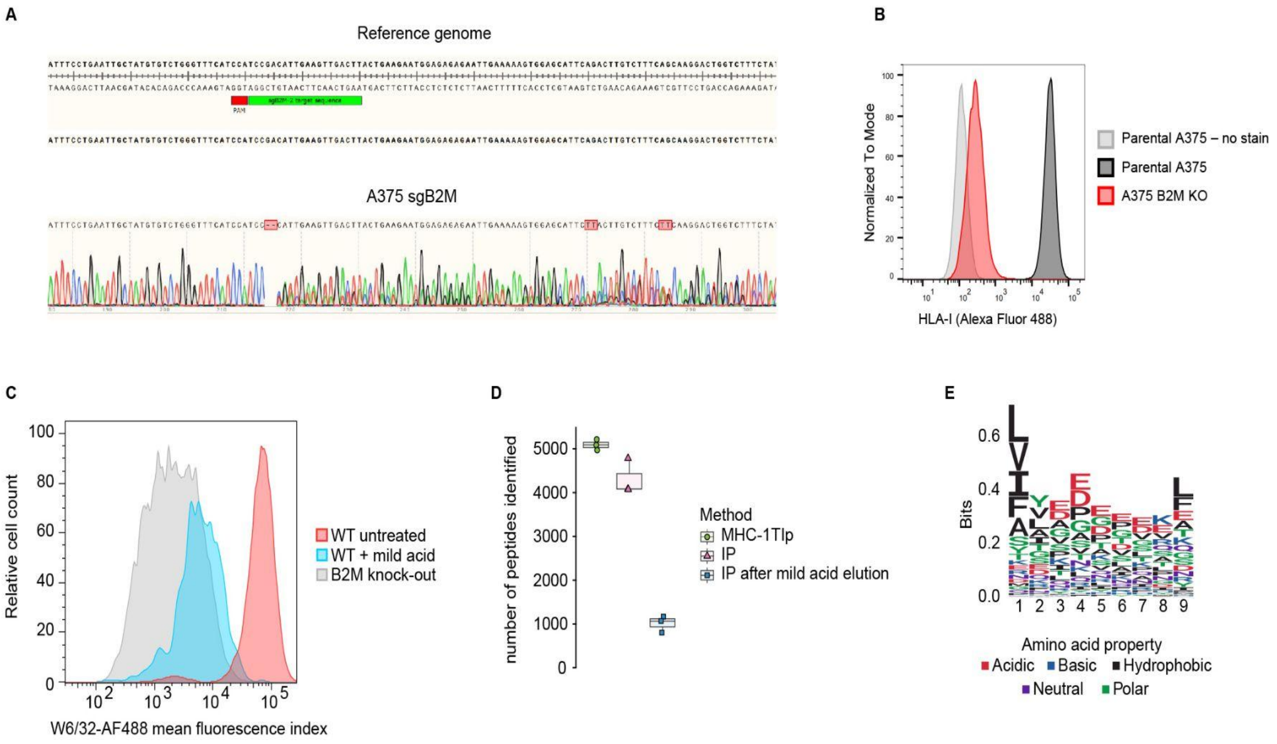

Supplementary Figure 1: Recovery of intracellular immunopeptides and dispase-derived peptides.

(A) Sanger sequencing Applied Biosystems Sequence Trace file alignment to sgB2M target sequence of the human reference genome hg38 in Snapgene v8.0.1.

(B) Flow cytometry staining with W6/32 antibody (pan-HLA-I)

(C) Flow cytometry staining with W6/32 antibody (pan-HLA-I)

(D) Number of immunopeptides identified with MHC1-TIP, immunoprecipitation (IP)-based immunopeptidomics and IP performed after mild acid elution in 10 million A375 cells

(E) Sequence motif generated from peptides of length 9, eluted from PDO-1 after dispase treatment to dissociate the organoids
