# Supplementary material for: MHC1-TIP enables single-tube multimodal immunopeptidome profiling and uncovers intratumoral heterogeneity in antigen presentation
Source: bioRxiv. 2025 Jul 21:2025.07.17.664894. Preprint. [Version 1] doi: 10.1101/2025.07.17.664894 (PMC12330465; doi:10.1101/2025.07.17.664894)
Supplement: Supplement 2 [file media-2.pdf]

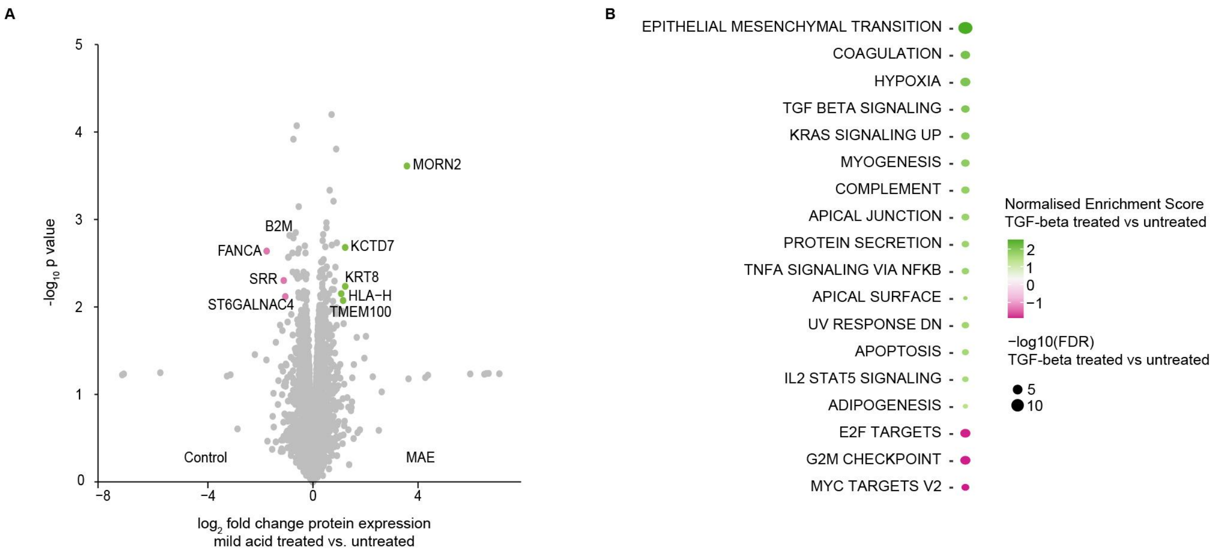

Supplementary figure 2: MHC1-TIP enables multi-omic profiling

(A) Changes induced in the proteome after mild acid elution. Green dots represent significantly upregulated proteins (FDR < 0.05 and log_2 fold change > 1) and pink dots represent significantly downregulated proteins (FDR < 0.05 and log_2 fold change < -1)

(B) Significantly enriched pathways (FDR < 0.05) after gene set enrichment analysis using Hallmark gene sets
