# Supplementary material for: MHC1-TIP enables single-tube multimodal immunopeptidome profiling and uncovers intratumoral heterogeneity in antigen presentation
Source: bioRxiv. 2025 Jul 21:2025.07.17.664894. Preprint. [Version 1] doi: 10.1101/2025.07.17.664894 (PMC12330465; doi:10.1101/2025.07.17.664894)
Supplement: Supplement 4 [file media-4.pdf]

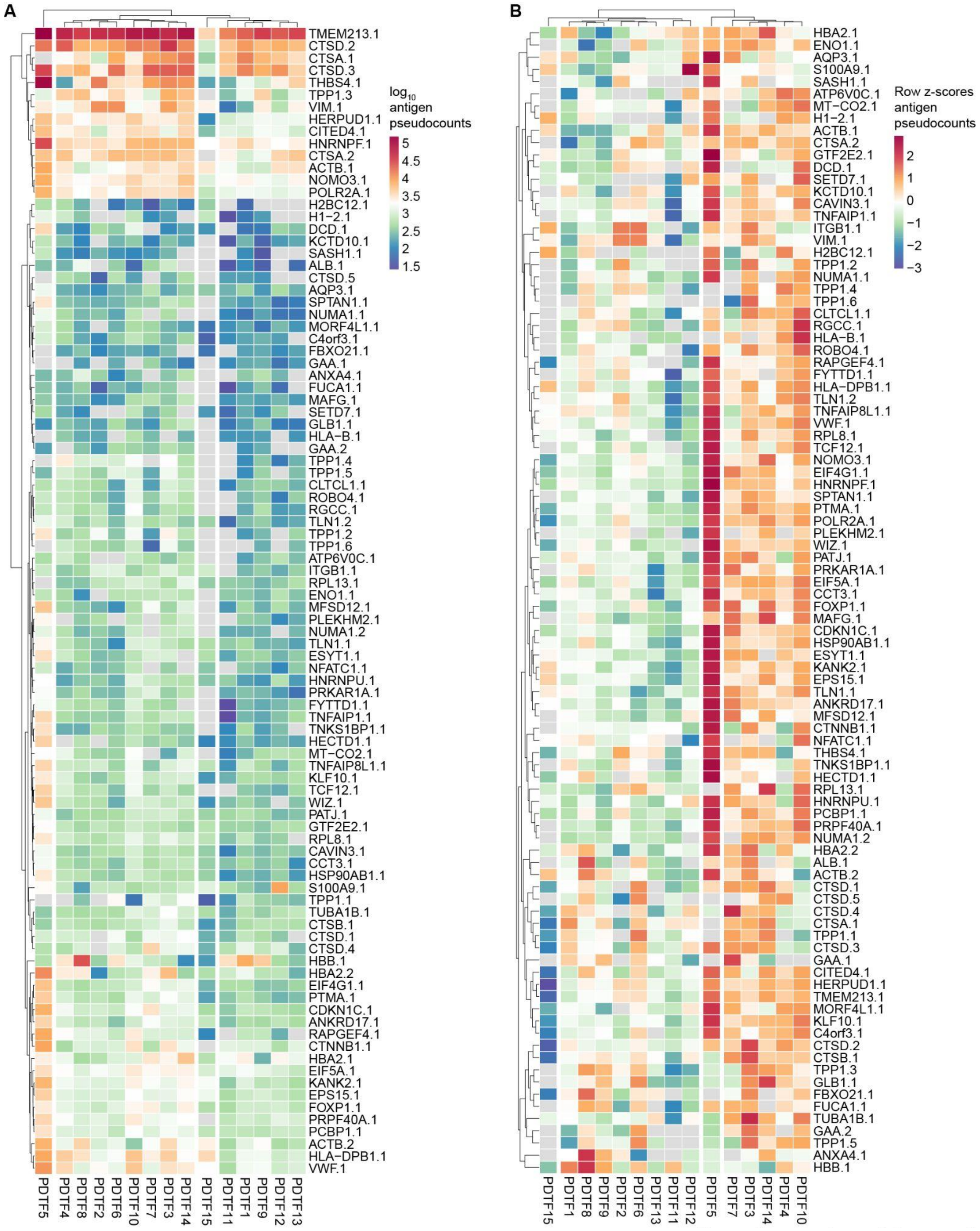

Supplementary Figure 4: Patient-derived tumour fragments show heterogeneity antigen abundances.

(A-B) Immunopeptides are labelled with the gene name of the parental protein of the antigen, conjugated with a numeric identifier for each peptide identified from that protein

(A) Heatmap displaying pseudocounts of antigen per cell in each tissue fragment. Detected antigen copy numbers per cell range 4 orders of magnitude.

(B) Scaling and centering antigen pseudocounts row-wise reveals heterogeneity in antigen presentation across the fragments
